# Oral Administration of Skin Gelatin Isolated from Chum Salmon (*Oncorhynchus keta*) Enhances Wound Healing in Diabetic Rats

**DOI:** 10.3390/md9050696

**Published:** 2011-04-26

**Authors:** Zhaofeng Zhang, Ming Zhao, Junbo Wang, Ye Ding, Xiaoqian Dai, Yong Li

**Affiliations:** Department of Nutrition and Food Hygiene, School of Public Health, Peking University, Beijing 100191, China; E-Mails: zhangzhaofeng@126.com (Z.Z.); zhaoming8210.student@sina.com (M.Z.); bmuwjbxy@bjmu.edu.cn (J.W.); dy03120319@163.com (Y.D.); daixiaoqian1985@gmail.com (X.D.)

**Keywords:** skin gelatin, wound healing, diabetic rats, angiogenesis

## Abstract

Care for diabetic wounds remains a significant clinical problem. The present study was aimed at investigating the effect of skin gelatin from Chum Salmon on defective wound repair in the skin of diabetic rats. Full-thickness excisional skin wounds were made in 48 rats, of which 32 were diabetes. The diabetic rats were orally treated daily for 14 days with skin gelatin from Chum Salmon (2 g/kg) or its vehicle. Sixteen non-diabetic control rats received the same amount of water as vehicle-treated non-diabetic rats. Rats were killed to assess the rate of wound closure, microvessel density (MVD), vascular endothelial growth factor (VEGF), hydroxyproline (HP) contents in wound tissues and nitrate in plasma and wound tissue at 7 and 14 days after wounding. Skin gelatin-treated diabetic rats showed a better wound closure, increased MVD, VEGF, hyproxyproline and NO contents and a reduced extent of inflammatory response. All parameters were significant (*P* < 0.05) in comparison to vehicle-treated diabetic group. In light of our finding that skin gelatin of Chum Salmon promotes skin wound repair in diabetic rats, we propose that oral administration of Chum Salmon skin gelatin might be a beneficial method for treating wound disorders associated with diabetes.

## 1. Introduction

Chronic wound healing is a troublesome and common complication of diabetes, resulting in significant clinical morbidity, such as nonhealing ulcers, infection, gangrene and amputation [1,2]. In recent years, treatment of diabetic wounds has intensified, but the amputation rate remains high and is associated with increased morbidity and mortality [3]. Thus, care for diabetic wounds remains a significant clinical problem and the development of therapies that improve wound healing in diabetic patients is of critical importance. However, due to toxicity or intolerance reactions in individual patients, therapeutic responses are limited in many diabetic patients despite the availability of new agents. Thus, compounds that provide greater symptomatic relief with less overall toxicity and minimal risks are preferred.

For the same reason, there is growing interest in gelatin as a therapeutic agent of potential utility in the treatment of wound healing for a high level of safety and overall lack of toxicity. Nowadays, over 250,000 metric tons of gelatin are produced worldwide every year, of which 60% is consumed by humans as a variety of products [4]. The authors also suggested that the unique amino acid and peptide profile of gelatin may be responsible for clinical observations supporting wound therapeutic efficacy. Since marine organisms comprise approximately one-half of the total global biodiversity, the sea is an enormous resource for novel compounds. Moreover, with recent advances in the high-tech instruments for isolation and characterization of marine natural products, there has been a growing interest in the importance of marine life as a source of new biologically active substances. Marine fish skin can serve as an additional source of proteins, especially gelatin. The uniqueness of gelatin from fish skin lies in its amino acid content. Although all gelatins are composed of the same 20 amino acids, there can be variation in the amount of amino acids, proline and hydroxyproline. Gelatin is effective in the treatment of wound healing based on clinical and animal studies [5–7]. Topical skin gelatin treatment has been proven to be effective for accelerating wound healing as part of photographic film [8,9]. However, it is not known whether oral administration of skin gelatin from Chum Salmon (*Oncorhynchus keta*) has enhancing effects on cutaneous wound healing in diabetic patients. Therefore, the purpose of the present study was to investigate the possible beneficial role of skin gelatin derived from Chum Salmon in an experiment model of type 1 diabetes-related wound healing disorders. These experiments could help to clarify the complex molecular interplay between wound healing and angiogenesis in diabetes and the possibility to prevent the cellular and molecular liabilities that hamper proper reparative angiogenesis.

## 2. Results

### 2.1. Animal Characteristics

The body weight and blood glucose levels of all animals at the time of wounding and at termination are shown in Table 1. Diabetic rats of both the vehicle- and skin gelatin-treated groups weighed significantly less at the end of the experiment compared to the day of wounding. Despite reduction of body weight, no difference was observed between the vehicle- and skin gelatin-treated groups over the process of the experiment. Blood glucose levels in diabetic rats used in the present study were consistently higher than 250 mg/dL and these levels were not changed by administration of skin gelatin from Chum Salmon.

### 2.2. Effect of Chum Salmon Skin Gelatin Treatment on Wound Closure

**Figure 1.**
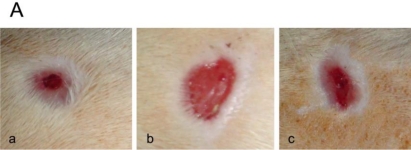

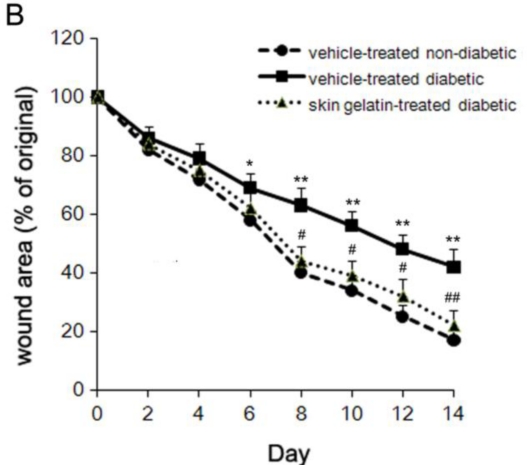
Effect of oral skin gelatin administration at the time of wounding on cutaneous wound closure rate in diabetic rats. (**A**) Representative photos of wounds in the non-diabetic and diabetic rats treated with either vehicle or skin gelatin at day 14 (**a**, vehicle-treated non-diabetic rats; **b**, vehicle-treated diabetic rats; **c**, skin gelatin-treated diabetic rats); (**B**) Group data of wound closure rate expressed as a percentage (%) of the initial wound size. The surface areas of the healing wounds were measured every 2 days and compared by one-way analysis of variance. * *P* < 0.05, ** *P* < 0.01—significance of the difference between the mean wound surface area of the vehicle-treated diabetic group and that of the vehicle-treated non-diabetic group. ^#^ *P* < 0.05, ^##^ *P* < 0.01—significance of the difference between the mean wound surface area of the skin gelatin-treated diabetic group and that of vehicle-treated diabetic group.

Firstly, the difference in wound healing between non-diabetic rats and diabetic rats was measured. As illustrated in Figure 1, the wound area of non-diabetic rats contracted almost to 17% of the original size by day 14 post-wounding, whereas diabetic rats showed significantly delayed wound healing: the wound area contracted to 42% of the original size by day 14. Next, the effect of skin gelatin treatment on wound closure in diabetic rats was investigated. The wound area seemed smaller in the skin gelatin-treated diabetic rats than in the vehicle-treated diabetic rats by day 7 and 14. In order to confirm this observation, the wound area was measured. The percentage of original wound area in the skin gelatin-treated diabetic rats was 16% smaller on day 7 and 35% smaller on day 14 compared to the vehicle-treated diabetic group.

### 2.3. Effect of Skin Gelatin on Wound MPO Activity

**Figure 2.**
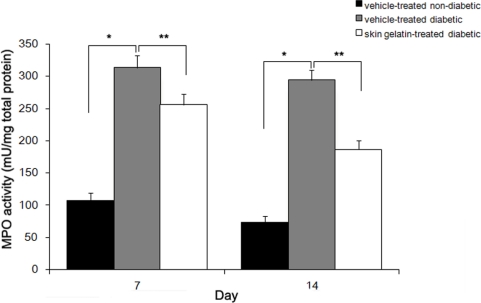
Effect of oral administration of skin gelatin on wound myeloperoxidase (MPO) activity in non-diabetic and diabetic rats at days 7 and 14. Results are mean ± SD, analyzed by ANOVA. * <0.05 different from same-day between two group. ** <0.01 different from same-day between two group.

Myeloperoxidase (MPO) activity in wounds from non-diabetic and diabetic animals was measured as an indicator of wound inflammatory activity. MPO activity was found to increase to an average of three and four-folds in vehicle-treated diabetic wounds by day 7 and 14 post-wounding compared with vehicle-treated non-diabetic animals, respectively. In vehicle-treated non-diabetic animals, wound MPO activity decreased with healing time. In the vehicle-treated diabetic wounds, the MPO activity remained significantly elevated at day 14. Skin gelatin treatment of wounds in the diabetic rats prevented this increase (Figure 2).

### 2.4. Histological Examinations

The histological examinations including the degree of fibroblast infiltration, collagen regeneration, vascularization and epithelialization in the wound area are shown in Table 2. Compared with vehicle-treated diabetic rats, a significant increase in the number of infiltrated fibroblasts in the subcutaneous tissue in the skin gelatin-treated diabetic group was seen by day 7 post-wounding (*P* < 0.05). Vascularization was significantly higher in the skin gelatin-treated diabetic rats than in the vehicle-treated diabetic group at 7 days (*P* < 0.05). Furthermore, significantly denser distribution as well as abundant collagen regeneration was observed in the skin gelatin-treated diabetic group at 7 and 14 days post-wounding (*P* < 0.05). Moreover, epithelialization was significantly greater in the skin gelatin-treated diabetic group than in the vehicle-treated diabetic group on day 14 (*P* < 0.05).

### 2.5. Collagen Accumulation

**Figure 3.**
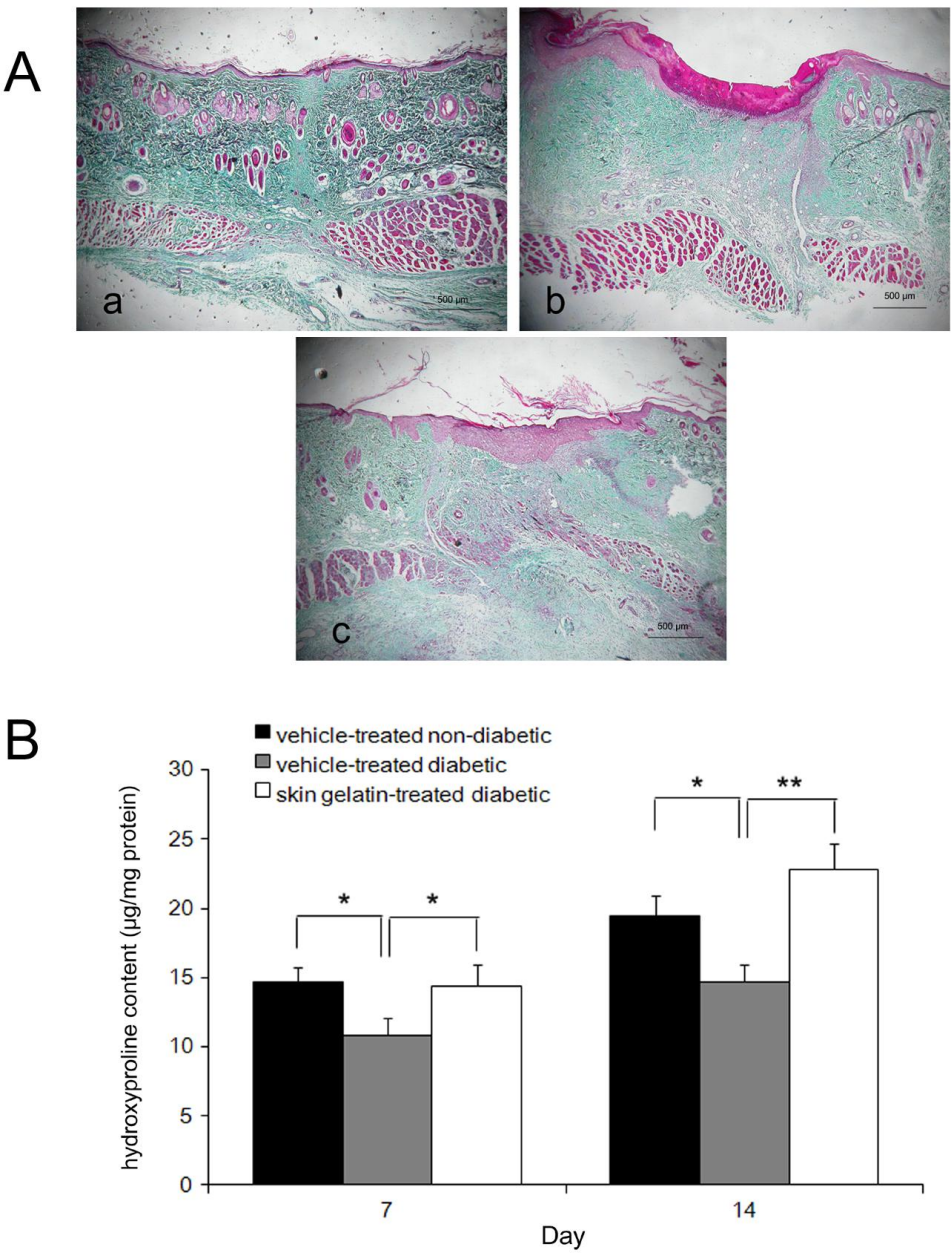
Collagen accumulation in wound areas of vehicle- and skin gelatin-treated non-diabetic or diabetic rats. (**A**) Representative high power view light micrographs (Masson’s trichirome staining) at day 14 (**a**, vehicle-treated non-diabetic rats; **b**, vehicle-treated diabetic rats; **c**, skin gelatin-treated diabetic rats); (**B**) Hydroxyproline levels in wound areas of the all treatment groups at day 7 and 14. Data are expressed as mean ± SD. * <0.05 different from same-day between two groups. ** <0.01 different from same-day between two groups.

Collagen deposition is an important event in the development of granulation tissue. Compared with the vehicle-treated diabetic group, the wound revealed a marked and robust increase in the organization of collagen fibers bridging the gaps in the skin gelatin-treated diabetic rats. Thus, collagen fibers were more organized and dense in the skin gelatin-treated diabetic group than in the vehicle-treated group (Figure 3A). To confirm these histological observations, hydroxyproline (HP) contents were measured in the lesions by 7 and 14 days after wounding. The results showed that hydroxyproline contents in the skin gelatin-treated diabetic group were higher than in the vehicle-treated diabetic group and significant differences were observed by 7 and 14 days post-wounding (*P* < 0.01) (Figure 3B).

### 2.6. Evaluation of Immunostaining for VEGF

**Figure 4.**
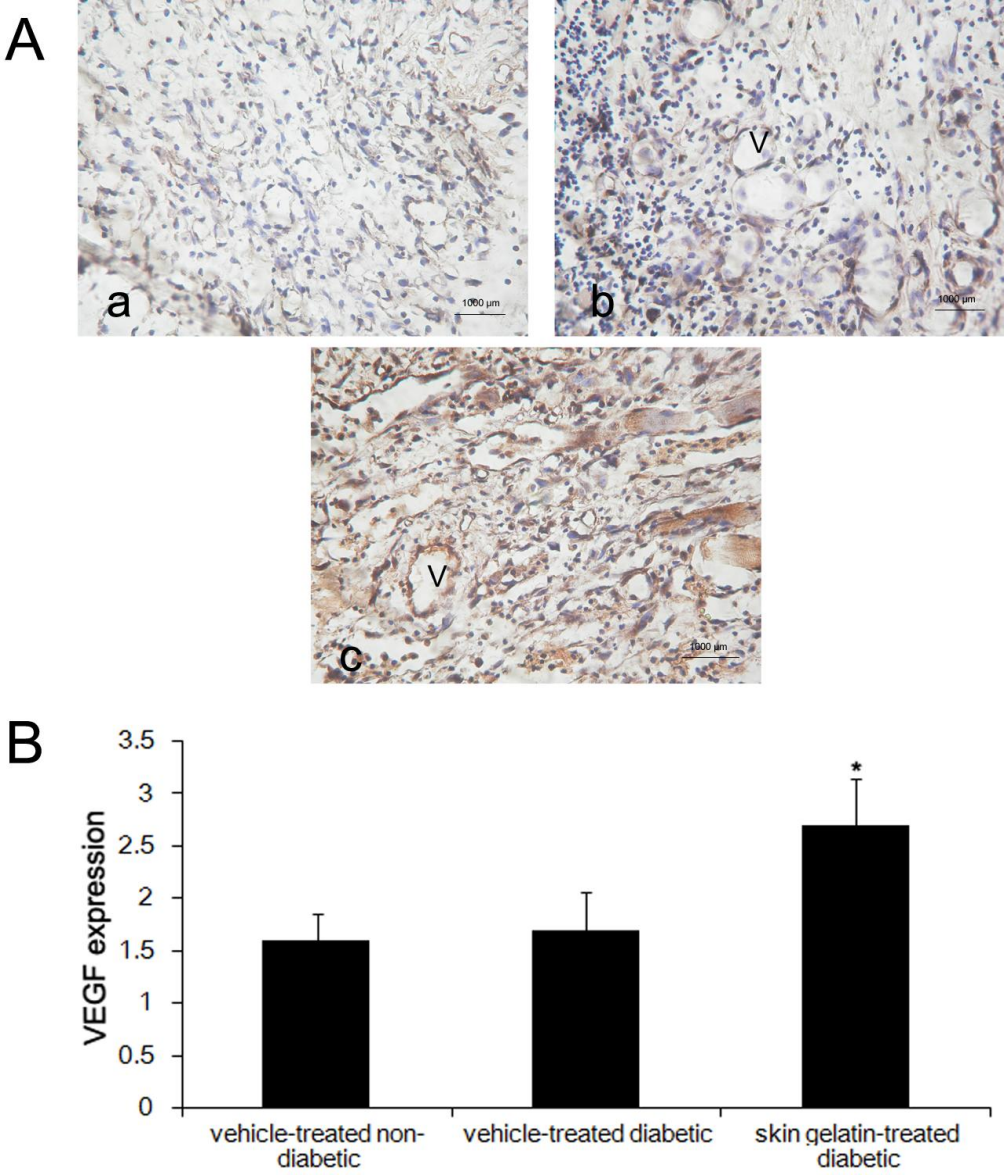
Expression of VEGF in wound tissue at day 14 after wounding in (**a**) vehicle-treated non-diabetic rats; (**b**) vehicle-treated diabetic rats and (**c**) skin gelatin-treated diabetic rats. Statistical analysis showed that the extent immunoreactivity of VEGF in the wound areas skin gelatin-treated diabetic rats is greater than that of the vehicle-treated diabetic group. * *P* < 0.05—significance of the difference between the mean VEGF in the wound area of skin gelatin-treated diabetic rats and that of vehicle-treated diabetic rats (magnification 200×; V, vessel).

The VEGF immunoreactivity of wound sections was examined in the skin gelatin- and vehicle-treated groups at 14 days post-wounding and the numbers of fibroblasts expressing VEGF were counted. As shown in Figure 4, VEGF immunolabeling was striking in the skin gelatin-treated diabetic rats, whereas it was faint in the vehicle-treated diabetic group. The numbers of migrated fibroblasts expressing VEGF in the skin gelatin-treated diabetic group were significantly higher than in the vehicle-treated diabetic group at 14 days post-wounding.

### 2.7. Effect of Skin Gelatin on MVD

**Figure 5.**
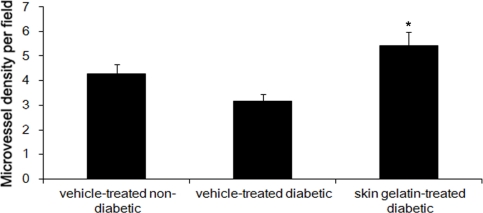
Oral skin gelatin treatment increases microvessel density (MVD) by 14 days after wounding by CD31^+^ cells counting. Statistic analysis showed that microvessel density in the wound areas of skin gelatin-treated diabetic rats is greater than that of vehicle-treated diabetic group. Each bar represents the mean MVD ± SD of the pooled data from each treatment group. * *P* < 0.05—significance of the difference between the mean MVD in the wound area of skin gelatin-treated diabetic rats and that of vehicle-treated diabetic rats.

After 14 days, the microvessel density (MVD) of the vehicle-treated wounds of diabetic rats was significantly lower than that of the vehicle-treated wounds of non-diabetic rats. When tissues from the vehicle-treated wounds of diabetic rats were examined histologically, there were avascular areas, ectasic vessels with edema and perivascular hemorrhage, and a marked reduction in capillary ramification. The MVDs in skin gelatin-treated wounds were significantly higher than that in the vehicle-treated wounds of diabetic rats (Figure 5).

### 2.8. Measurement of Nitrite Levels

**Figure 6.**
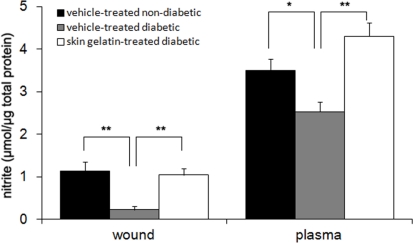
Nitrite levels in wounds and plasma of the non-diabetic and diabetic rats treated with either vehicle or skin gelatin at day 14 after wounding. Data are expressed as mean ± SD. * *P* < 0.05 different from same-day between two groups. ** *P* < 0.01 different from same-day between two groups.

Nitrite dosage was used as an index of NO synthesis, because nitrite is a stable molecule and accounts for more than 90% of total measurable nitrite and nitrate. Fourteen days after wounding, the nitrite levels were respectively 5-fold and 1.7-fold greater in the skin gelatin-treated group than in the control group in both the wound and plasma (Figure 6).

## 3. Discussion

The present study presented that oral administration of skin gelatin from Chum Salmon accelerates cutaneous wound healing in the skin of diabetic rats.

The inability of wounds to heal in diabetes mellitus patients is associated with an abnormality in one or more phases of the healing process. In our study, we observed that defective wound repair in diabetic rats is associated with reduced wound vascularization and collagen synthesis, and with an increased extent of inflammatory cells in wound tissues.

In acute wound healing, the inflammatory response should occur rapidly and sustain for 3 days to permit the development of subsequent phases of wound healing [10–12]. This requires that inflammatory cells (such as neutrophils and macrophages) migrate to the wound area and phagocytize necrotic tissue and microorganisms. However, the inflammatory response in chronic wound healing, such as diabetes, will last for extended periods and affect wound regeneration. To evaluate the inflammatory infiltrate, MPO activity, a better indicative of the inflammatory activity in the granulation tissue, was measured 14 days after wounding. The inflammatory response was higher in the diabetic group until 14 days after wounding in the present study. Skin gelatin administration significantly reduced the inflammatory response and increased the quantity of fibroblast cells in the wound area of diabetic rats when compared to vehicle-treated diabetic group. The positive effect of skin gelatin on the inflammatory response of diabetic lesions may be explained by skin gelatin-stimulated NO synthesis, which was consistent with previous work [13,14]: NO may directly stimulate the production of cytokines of inflammatory cells, consequently accelerating the development of the subsequent phases of wound healing.

Wound closure involves a complex and superbly orchestrated interaction of cells, extracellular matrix, and cytokines. The increased rate of wound closure in skin gelatin-treated wounds might be attributed to increased proliferation and transformation of fibroblast cells into myofibroblasts. Histological findings also showed enhanced proliferation of fibroblasts and re-epithelialization in skin gelatin-treated wound tissues of diabetic rats. The early re-epithelialization and faster wound closure in skin gelatin-treated wounds might also be associated with increased keratinocytes proliferation, and their migration to the wound surface [15,16].

The fibroblasts are responsible for the synthesis, deposition, and remodeling of the extracellular matrix. After migrating into wounds, fibroblasts initiate the synthesis of the extracellular matrix. Collagen is a major protein of the extracellular matrix and is the component that ultimately contributes to wound strength [10]. The enhanced level of hydroxyproline in skin gelatin-treated diabetic rats probably provides the strength to the regenerated tissue.

Angiogenesis during wound repair serves the dual function of providing the nutrients required by supply essential nutrients and oxygen to the wound site, and promoting granulation tissue formation [11,17,18]. In the present study, skin gelatin was found to increase angiogenesis as evidenced by MVD. Histological evaluation also revealed increased blood vessel formation in the granulation tissue of skin gelatin-treated diabetic rats. Enhanced expression of VEGF as revealed through immunohistochemistry in skin gelatin-treated diabetic rats as compared with vehicle-treated diabetic group might be responsible for this activity. In fact, it is well-known that vascular endothelial growth factor (VEGF) produced by fibroblasts is one of the most potent angiogenic cytokines [19,20]. VEGF appears to be a key factor in pathological situations such as tissue repair, which involves neovascularization and increased vascular permeability. VEGF improves angiogenesis during the process of wound healing by stimulating the migration of endothelial cells through the extracellular matrix [21]. In agreement with our present finding, Galiano *et al.* [22] demonstrated that VEGF local therapy in db/db mice enhanced neovascularization at the wound site through a stimulation of local angiogenesis, thus suggesting that VEGF or agents stimulating its production may be exploited to promote tissue repair in a wide variety of acute and chronic injuries, particularly in conditions such as diabetes or aging [23]. However, it seems that VEGF staining is much lower in control non-diabetic rats than in vehicle treated diabetic rats. It could be interesting to compare at an earlier stage, for example day 7, since at day 14 the granulation tissue of non diabetic wounds is more mature and the signal could be lower.

In the present study, skin gelatin administration enhanced blood vessel density in excisional lesions of diabetic rats when compared to the vehicle-treated diabetic group. The beneficial effect of skin gelatin on angiogenesis in diabetic lesions may be explained by the increased NO synthesis. It has been shown that NO increases VEGF expression, which is the most potent angiogenic factor during wound healing [14], thereby, stimulating the formation of new blood vessels [24]. Thus, we suggest that skin gelatin treatment may result in enhanced angiogenesis through its stimulatory effects on NO synthesis leading to improved formation of granulation tissue.

In addition, there is abundant evidence suggesting that oxygen free radicals play an important role in diabetic wounds. These molecules cause failure of diabetic wound healing; therefore, antioxidants partly improve the healing in diabetic skin wounds [25]. It was also found that in some injury cases ointment contains superoxide dismutase (SOD), which stimulates wound healing. Our previous study and other studies have demonstrated that marine fish skin gelatin has potent antioxidant and free radical scavenging effect [26–28]. Hence we speculated that skin gelatin from Chum Salmon enhanced induction of antioxidant levels at an initial stage of healing, which may be an important contributory factor in the healing property.

## 4. Methods

### 4.1. Preparation of Skin Gelatin

Gelatin was derived from the skin of wild-caught Chum Salmon (*O. keta*) (average body weight, 1.47 kg) and donated by CF Haishi Biotechnology Ltd. Co. (Beijing, China). The procedure of skin gelatin preparation and identification is according to the method described previously [29,30]. In brief, after descaling, Chum Salmon skin was washed with running tap water for 1 h. It was then soaked in 0.4 (w/v) NaOH and HCl aqueous solution for 4 h at room temperature, respectively. The skin was washed again with running tap water until pH was neutral. Finally, the skin was extracted with distilled water for 1.5 h at 70 °C. The extract was filtered through two layers of cheese cloth and evaporated at 70 °C to remove 70% of water. The filtrate was dried in a hot-air oven at 50 °C for 18 h. The resulting gelatin was stored in a desiccator for further use.

### 4.2. Formation of Diabetic Rats

All experimental procedures in this study were in accordance with the guidelines prepared by the Animal Care and Committee of Peking University Animal Center. Forty-eight male Sprague-Dawley rats weighting 250–300 g were housed individually in rooms maintained under controlled environmental conditions (12-h light/dark cycle, temperature approximately 25 °C). The rats were acclimated for 1 week before the study and had free access to standard laboratory chow and water *ad libitum*. Diabetes was induced by a single 45 mg/kg intraperitoneal injection of streptozotocin (Sigma, St Louis, MO, USA) in saline-sodium citrate buffer. Blood glucose levels were measured using a rapid glucometer. Ten days after streptozotocin injection, thirty-two animals with blood glucose levels above 250 mg/dL were defined as diabetic and used in the study. Sixteen healthy rats were used as the vehicle-treated non-diabetic control group.

### 4.3. Full-Thickness Skin Wound Preparation

Ten days after streptozotocin administration, all rats were anesthetized with an intraperitoneal injection of sodium pentobarbital (45 mg/kg body weight). The dorsal region was shaved with an electric clipper and the surgical area was disinfected with 70% alcohol. A sterile template of 2.0 cm in diameter was placed on each side of the mid-back and a full-thickness wound to deep fascia corresponding to the template was made by excising the skin. The wounding day was considered as day zero. Food consumption, body weight and blood glucose levels were recorded on the day of wounding, and thereafter weekly until euthanasia.

The 32 diabetic rats were then divided randomly into 2 groups: skin gelatin-treated diabetic group (*n* = 16), which received skin gelatin from Chum Salmon, 2 g/kg·bw, dissolved in water; and vehicle-treated diabetic group (*n* = 16), which received water (in the same amount as the experimental group). The non-diabetic control rats (*n* = 16) received the same amount of water (vehicle-treated non-diabetic group). Oral administration of skin gelatin began on the wounding day and was maintained daily until to sacrifice.

### 4.4. Wound Closure Measurement

The edge of migration epithelium was easily discernible from the moist granulation tissue and the presence of the epithelial border was the edge of the healing wound. The edge of the wound was traced onto transparent paper every 2 days and the wound area was determined by planimetry using Imagemeasure. The trace taken immediately after wounding was used as the original area (day 0 area).

### 4.5. Histological Analysis

After the final wound tracing on either day 7 or day 14, eight rats of each group were given a lethal ethylether inhalation, and the entire wound, including a 5 mm margin of unwounded skin, was excised down to the fascia. The wound was divided in half through the least healed portion. One-half of the wound was stored in the liquid nitrogen for future molecular studies, the other half was fixed in 10% neutral-buffered formalin for at least 24 h, followed by processing for conventional paraffin embedding. 5 μm-thick sections were mounted on glass slides, dewaxed, rehydrated to distilled water, and stained with hematoxylin and eosin (*H&E*) or Masson’s Trichrome. As part of the histological evaluation, all slides were examined by two pathologists, without knowledge of the previous treatment, under a microscope from 20× to 100× magnification. Sections were semi-qualitatively assessed under the light microscope and observed in respect of fibroblast proliferation, collagen formation, angiogenesis and epithelialization using a 4-point scale as follows: (0: none; 0.5: few; 1: moderate; 2: many and 3: considerable).

### 4.6. Immunohistochemistry

Paraffin-embedded tissues were sectioned (5 μm), and antigen retrieval was performed using in 10 mM sodium citrate buffer. Endogenous peroxidase activity was blocked by treating sections with 0.3% hydrogen peroxide in methanol for 15 min. Tissues were treated with polyclonal rabbit anti-VEGF and anti-CD31 antibody (Santa Cruz Biotechnology, Inc., USA; dilution 1:300) overnight at 4 °C. Specific labeling was detected with a peroxidase-conjugated goat anti-rabbit IgG and avidin-biotin peroxidase complex. Slides were then mounted with coverslips and analyzed by two blinded pathologists. Assessment of immunoreactive cell density in the tissue was performed and graded semi-quantitatively as absent (0), few (1), moderate (2) or numerous (3).

### 4.7. Assess of MVD

Microvessel density (MVD) in the wound granulation tissues was assessed as a parameter of wound-induced angiogenesis in a blind manner according to the established methods described previously [31]. The expression levels of CD31 antigen can be used to assess MVD in healing wounds. MVD was determined in five regions of interest in each specimen in which the CD31 antibody signal was the moist intense. The number of blood vessels was counted under a light microscope by two investigators who were blinded to the treatment of the rats. Microvessel density was expressed in terms of microvessel number per observed area (vessel number/mm^2^).

### 4.8. MPO Activity Assay

To estimate the number of neutrophils in the wounded area, myeloperoxidase (MPO) activity in wound lysate was assayed according to Stark and collaborators with minor modifications [32]. An aliquot of wound lysate was mixed in phosphate buffer (80 mM) containing 0.5% hexadecyltrimethyl ammonium bromide at pH 5.5. The mixture was centrifuged at 12,000 rpm for 15 min to extract the MPO. Subsequently, the supernatant was mixed with 3,3′,5,5′-tetramethylbenzidine dihydrochloride (1.9 mg/mL) and hydrogen peroxide. Thereafter, the mixture was incubated at 37 °C for 15 min and then mixed with acetate buffer pH 3.0. The developed color was read spectrophotometrically at 630 nm. MPO concentrations in the sample were determined from a standard curve generated by different concentrations of MPO from human leukocytes. Data are expressed as mU MPO per mg total protein.

### 4.9. Determination of Nitrite Concentration

Levels of nitrite in wound lysates and plasma were determined by a spectrophotometric method based on the Griess reaction. An aliquot of each sample (100 μg) was mixed and incubated with 100 μL of Griess reagent at room temperature for 10 min. Thereafter, the developed color was spectrophotometrically read at 557 nm. Nitrite concentrations in the samples were determined from a standard curve generated by different concentrations of sodium nitrite. Data are expressed as μmol nitrite per μg total protein.

### 4.10. Determination of the Hydroxyproline Content in the Wounds

Full thickness skin samples taken from the excision were weighed and kept at −20 °C in physiological saline. To eliminate water, a 100 mg sample of excision wound tissue from each dead rat were kept at 60 °C for 72 h in a drying oven and were weighed again. Dry and defatted tissue was hydrolyzed in 6 M HCl for 18 h at 110 °C. Hydrolysate was then diluted with distilled water, neutralized with 6 M NaOH and centrifuged at 3000 rpm for 15 min. Hydroxyproline levels were measured in this hydrolysate as previously described [15]. The difference between wet and dry weights was taken as water ratio.

### 4.11. Statistical Analysis

Data for each study parameter from the skin gelatin- and vehicle-treated wound tissues for each group were presented as mean ± standard deviation. Data from each group were statistically analyzed by one-way analysis of variance, except the data obtained from the semi-quantitative analysis using Kruskal-Wallis test. Differences were considered statistically significant when *P* < 0.05.

## 5. Conclusions

In conclusion, oral skin gelatin administration improves cutaneous wound healing of hyperglycemic diabetic rats by reducing inflammatory response, improving wound contraction, collagen deposition and angiogenesis, and stimulating NO synthesis. Taken together, all evidence above leads us to propose that oral administration of skin gelatin from Chum Salmon might be a beneficial method for treating wound disorders associated with diabetes.

## Figures and Tables

**Table 1.** Effect of oral skin gelatin treatment on body weight and blood glucose levels in the three experimental groups.

	**Vehicle-treated non-diabetic (*n*= 8)**		**Vehicle-treated diabetic (*n*= 8)**		**Skin gelatin-treated diabetic (*n*= 8)**		***P*-value (between groups after treatment)**
	**Day 0**	**Day 14**	**Day 0**	**Day 14**	**Day 0**	**Day 14**	
Body weight (g)	263 ± 11	297 ± 13	248 ± 10	232 ± 16	250 ± 12	234 ± 14	<0.05
Blood glucose levels (mg/dL)	115 ± 6.3	119 ± 5.8	398 ± 26	384 ± 23	402 ± 27	389 ± 28	<0.05

Values are presented as mean ± SD. Statistical significance is set at *P* < 0.05.

**Table 2.** Histological scorings.

	**Fibroblasts**	**Vascularization**	**Collagen**	**Epithelialization**
Day 7				
Vehicle-treated non-diabetic	1.4 ± 0.2	1.2 ± 0.1	1.7 ± 0.1	1.8 ± 0.2
Vehicle-treated diabetic	1.3 ± 0.2	1.2 ± 0.2	0.9 ± 0.2 **	1.2 ± 0.3 **
Skin gelatin-treated diabetic	1.8 ± 0.2 ^#^	1.4 ± 0.1 ^#^	1.3 ± 0.1 ^#^	1.5 ± 0.2 ^#^
Day 14				
Vehicle-treated non-diabetic	1.8 ± 0.2	1.9 ± 0.1	1.9 ± 0.1	2.6 ± 0.3
Vehicle-treated diabetic	1.7 ± 0.2	1.6 ± 0.2 *	1.2 ± 0.1 **	1.8 ± 0.2 **
Skin gelatin-treated diabetic	2.3 ± 0.2 ^#^	2.3 ± 0.2 ^#^	1.5 ± 0.2 ^#^	2.2 ± 0.2 ^#^

Note: Histological scorings were performed among the treatment groups. Data is expressed as mean ± SD.

* *P* < 0.05,

** *P* < 0.01—significance of the difference between the vehicle-treated diabetic group and the vehicle-treated non-diabetic group.

# *P* < 0.05—significance of the difference between the skin gelatin-treated diabetic group and the vehicle-treated diabetic group.
